# The Virginia Springs, and Springs of the South and West

**Published:** 1859-07

**Authors:** 


ART. III.—*The Virginia Springs, and Springs of the South and West.* By J. J. MOORMAN, M.D., Resident Physician at the White Sulphur Springs. With Maps and Plates, and the Routes and Distances to the various Springs. 12mo., pp. 403. J. B. Lippincott & Co.: Philadelphia, 1859.

*A Treatise on Baths; including Cold, Sea, Warm, Hot, Vapor, Gas, and Mud Baths: also on Hydropathy and Pulmonary Inhalation; with a Description of Bathing in Ancient and Modern Times.** By JOHN BELL, M.D., etc. etc. Second edition. 12mo., pp. 658. Lindsay & Blakiston: Philadelphia, 1859.

HYDROLOGY, in its application to hygiene and therapeutics, is beginning to acquire that importance to which it is so eminently entitled. To our thinking, it is time that the virtues of water, both in its internal and external use, for the preservation of health and the cure of disease, should receive its proper share of consideration. A thorough study and due appreciation of the subject will, we are convinced, go farther than any other course of investigation and analysis to simplify the practice of medicine, while pathology, in its large and proper sense, will still receive its full share of attention. As has been said by an author familiar with the subject:—

> Gifts freely dispensed are seldom appreciated at their just value or received in a suitably thankful spirit. The air which we breathe, and by which we live, would seem to be regarded by civilized man as something intrusive,

* The superscription to the title conveys an apothegm—Water as a Preservative of Health, and a Remedy in Disease.

if not positively noxious, which must be carefully shut out from his habitation, and from all the edifices in which he meets his fellow-men for the purposes of religious worship, of commerce, of amusement, of law and legislation. The water, which in a state of vapor fills and makes up so much of the atmosphere, and which in its fluid state is both food and the vehicle of all that is nutrimental in organized beings, and the imbibition of which by them is a necessary condition for their growth, maturity, and fruition, is too often looked at askance, by the same civilized man who takes all possible pains to alter the properties, while impairing the purity of this universal fluid and menstruum, by mixing it with the distillations of art, and the products of the vat and the wine press, not to speak of the so-called liquors and cordials compounded of all these, and as if devilish ingenuity was still unsatisfied, drugged with recognized poisons of every kind. On the same line of gifts beneficently bestowed on man, but either held at a cheap rate, or neglected by him, are Mineral Waters, which jet out from nearly all parts of the earth's surface; compounds, often of the most varied chemical elements, prepared in Nature's Laboratory and Nature's Pharmacy, ready for use, and adapted to the cure of multifarious diseases, as simple water is to the preservation of health. What a contrast between, on the one hand, the alcoholic and narcotic adulterations of water, to gratify a perverted appetite, and to excite and so generally derange the functions of the organism; and, on the other hand, the addition of earthy and mineral salts which the water receives in its percolation through different strata; and thus when it gushes up as a spring, it is found to be endowed with sanitary and therapeutic virtues, so great and so diversified, that it has sometimes been said, a region of mineral springs is a region of miracles! Equally striking is the difference between the destructive distillation of the nutritive cereals and fruits into ardent spirits, and the conservative distillation of simple water, when in its deep, subterraneous course among primitive rocks it is subjected to central and volcanic fires, and converted into vapor, which, ascending from this great natural alembic, is condensed in the upper strata into thermal water, and a little later becomes in this state a gushing thermal spring. For the most part, the water, if of a temperature so elevated as to bring it under the category of a warm or of a hot spring, is but slightly mineralized, and it is sometimes more exempt from foreign substances than even the water of common springs in the same region of country.

It is with mineral waters as with most of the articles of the Materia Medica. Their remedial virtues were known as matters of experience long before the basic elements and proximate principles were discovered. Chemical analysis, which has revealed to us the intimate composition of these waters, is, if it be viewed as a means of guiding us to an acquaintance with their therapeutical use, secondary in the order of time as it is in the scale of experimental observation. It may confirm, but does not originate our belief, and, at the best, it can only be regarded as suggestive, in as far as it exhibits in the water of a recently discovered spring, a medicinal substance or medicinal substances identical with those in the water of another spring, the curative powers of which have been ascertained by long experience. A knowledge of the chemical constituents of a mineral water is also suggestive in another way, by leading the physician to more diversified clinical trials of the curative effects of the water than would otherwise have been attempted. Chemistry, in its suggestive relations to the therapeutics of mineral waters, enables us to improve our imperfect knowledge of those of our own country, by comparing them with those of Europe in a more precise and positive manner than could be done were we to rest the comparisons merely on their sensible properties. Thus, for example, from the long and fully recorded experience of the medicinal virtues of any one chemical division of the mineral waters of England, France, Germany, or Italy, we may obtain, if not rules, at least pregnant hints, to aid us in directing the use of waters of a similar chemical division in the United States. But even here we may be misled by apparent resemblances between two waters, which have an active principle—sulphur or iron, for instance, common to both, but which may differ materially in the number and proportion of their saline constituents. This circumstance must be continually borne in mind in the classification of mineral waters, which, like all classifications, is, at the best, but approximative, either as regards resemblances in the characteristics of the individual ones which, grouped together, constitute the class, or the differences which separate them from another group and another class.

The chemical hypothesis of the operation of mineral waters offers an explanation of some of their effects, as evinced in a change of the fluids of the organism. Lithic acid is neutralized by water holding in solution a large proportion of an alkali, and the predominance of this acid in the urine is destroyed, and the alkaline is substituted for the acid reaction, by the use of such a water as that of the Vichy Springs. Demonstrations of this kind do not, however, occur in such number and variety as to authorize a belief in a constant and intimate relation between the chemical constitution of a mineral water, and its chemical action on the organs, with the effect of restoring these latter to the healthy exercise of their functions. In these cases it must be remembered that the removal of a symptom does not necessarily imply the removal of the disease, nor even always the cause of the symptom itself. There is something in the mode of combination of the several constituents of mineral waters, which gives them a therapeutic power not to be measured nor ascertained by an analysis and exhibition of each separate constituent or element. The active principle in a water resembles vitality in the organism, in its being only known by its effects. Looking at the subject in this light, Chaptal might well say, that "chemists only analyze the cadaveric remains of mineral waters." Their life and their spirit disappear in the process of analysis. We must continue, notwithstanding, to look hopefully to chemistry, not indeed for a solution of the problem, but for an increase of the facts which give collateral aid to our causative researches into the subject.*

Analyses may furnish general indications, but they fail to enable us to distinguish the characteristic shades of difference among springs of even the same class. The best, and indeed the only satisfactory means of ob-

* On the Therapeutical Value of the Waters of Mineral Springs. By John Bell, M.D.—*Am. Med. Gaz.*, Jan. 1859.

taining an accurate acquaintance with the therapeutical effects of mineral waters, is by carefully made clinical records of cases which were treated by administering them on the spot. Opportunities of this kind were first furnished in France, two centuries and a half ago, by edicts of Henry IV., whose early life was passed in the region of the springs of the Pyrenees. He appointed officers who were charged with the supervision of the baths and mineral springs of the kingdom. His edicts on this subject were confirmed by Louis XV. and Louis XVI. The properties of these waters were studied, by some under the promptings of a scientific zeal and in a spirit of philanthropy; by others, with the hope of finding a remedy for certain diseases of royal personages. Fagon, physician to Louis XIV., examined with care the waters of Bonnes and Barreges, in order to ascertain whether they would be adapted to the cure of the fistula with which that monarch was afflicted. Chirac directed his attention to the waters of Balarac, and sought in them a remedy for the wound of the Regent, the Duke of Orleans.

A still more formal and methodical plan of investigation was set on foot, by the appointment of resident medical superintendents, on whom it was enjoined to keep a regular record of the cases of those invalid visitors at the springs who came under their charge. By a royal decree, issued in 1823, the medical inspectors of the several springs were required to send, each year, to the minister of agriculture and commerce, a report of the diseases which they had observed and treated, or seen treated, during the season for the use of the waters. In order, however, to be able to discharge their duty in this respect, the concurrence or preliminary aid of the medical advisers of the invalids, in their respective homes, is necessary, so that they should be informed of the antecedent history, both of the character and progress of the disease, and of its therapeutical treatment. Often the malady persists during the period of the use of the waters, or the season, as it is called, of three or at most four weeks duration, and it may, on the return home of the patient, become aggravated, or be amended or cured; circumstances these, which put it out of the power of the medical inspector, or other resident physician, to complete the history of the cases of those who resort to the springs, for the purpose either of drinking its water or using it as a bath, or often of employing it in both ways. Then again, many follow the directions, in these respects, of their own professional advisers; while others, after having consulted one or more physicians, follow ultimately their own notions.

Of 4500 persons who took the baths at Rennes, in the years 1847, 1848, and 1849, we are told by M. Cazaintre, the medical inspector, that he was called on to see only 611 out of this whole number. It is a lamentable thing, writes M. C., in his report, to see numbers of persons take the baths and drink the mineral waters to excess, without considering the nature or stage of their malady, or the susceptibility of their organism. Hence, in this irregular condition of things, some take the baths who ought only to drink the waters, and others drink the waters who ought only to bathe; a third party, in fine, have recourse to both baths and drinking the water, and others in abstaining rigorously from all. How often, continues M. C., has it not happened to me to send away patients who labored under hypertrophy of the heart, or who had reached the last stage of pulmonary consumption, or who would have inevitably perished in the bath from a stroke of apoplexy, if chance or an aggravation of their sufferings had not made me cognizant of their condition, and enabled me in time to forbid all thermal treatment! In view of these abuses and evils, the Commission on Mineral Waters, appointed by the French government, in its report for the years 1849 and 1850, recommends strongly such stringent regulations as would prevent any person from making use of mineral waters, without the authorization of the superintendents of mineral springs, or the prescription of some other physician.

The preceding picture falls short of representing the state of things at the watering places in the United States, all of which are without medical superintendents, and not a few are destitute of resident physicians; and at which, more than in France, the visitors acting, we must suppose, under certain notions of their reserved rights, to do what they like with their own, or, more probably, without a definite notion of any kind, commit all sorts of extravagances in bathing in and drinking the waters of mineral springs.

These observations on the means of ascertaining the medicinal virtues of mineral springs and of the steps elsewhere taken to reach positive conclusions on this point, as also a notice of the impediments offered by the ignorance of the invalids, who are the parties most interested in a solution of the problem, cannot be regarded as an inappropriate introduction to a rapid analytical sketch of the new edition of Dr. Moorman's Treatise on the Virginia Springs, which now includes a succinct account of the Springs of the South and West. This gentleman, as we learn from his preface, has directed his special attention for more than twenty years to the investigation of the nature and medicinal application of mineral waters. During this time he has resided throughout each season, in which the waters were used, at the White Sulphur Springs, where, in the character of resident physician, he has enjoyed ample opportunities of witnessing the various and modified effects of the water, in almost every variety of disease and state of the system. But, although his headquarters have been at the springs just named, he has not neglected the others, which are so bountifully spread over the southwestern region of Virginia.

Of them he speaks appreciatively, both from the results of his own occasional visits, and, still more, from the medical and other testimony of persons on the spot.

Looking to the fact of the existence of the valuable mineral waters of *Capon* and *Berkeley*, at the northern, and the recently discovered waters at the southern extremity of the line of our great Apalachian chain, the Virginia Spring Region must, Dr. Moorman thinks, be looked on as occupying a larger area than has hitherto been supposed, and as embracing the entire length of the Alleghany, from the Potomac to the extreme southern terminus. "Errors in Spring Nomenclature" are pointed out by the author, as in the instances of the so-called alum waters, the efficacy of which depends not merely on the presence of this salt, but on its association with other salines, giving a resulting compound *sui generis;* and the sensible effects of which, in their acting as a mild purgative, for example, are quite different from those of alum or of a simply aluminous water. So also in the terminology of various sulphur springs derived from the presumed color of the waters; and hence the designations of *White, Red, Blue, Gray, Black, etc.* "convey no idea at all of the quality or appropriate medical applicability. The natural deposits of all sulphur waters, uninfluenced by foreign bodies, are essentially of the same color, being white or opake-white; the various shades of red, blue, gray, black, etc. are occasioned by the influence of light or shade, or by chemical changes produced by contact with foreign bodies."

In the chapter given to the consideration of mineral waters in general, the author deplores the want of information, both among physicians and the public at large, in regard to this class of medicinal agents, and the mistakes and injuries thence resulting. Remarks are made on the *modus operandi* of mineral waters, their alterative action, their inapplicableness to acute diseases, and their adaptation to chronic ones. In giving place to the following remarks, we believe that we shall express the views of Dr. Moorman as we certainly shall our own on these points:—

Mineral waters are, for the most part, exciting; and hence they are contra-indicated in acute diseases, and in those marked by much irritation or excessive excitability. They are illy adapted to persons of a sanguine temperament or a plethoric habit, and to those liable to cerebral congestion or to hemoptysis. How often does it not happen that judicious resident physicians at the springs are obliged to send away patients suffering from hypertrophy of the heart, or aneurism of this organ and of the great vessels, or who have reached the last stage of phthisis! Physicians do not always seem to consider, that chronic disease, which has been intractable to a great variety of treatment, and is regarded as incurable, is therefore a fit subject for all kinds of experiments. That which is bad must not become worse under our hands—as when a chronic is converted into an acute inflammatory disease, and that which might have dragged its slow length along for years, is suddenly stopped short by death. The plea of giving the patient an additional chance of cure by sending him to watering places, either for the purpose of drinking mineral waters or of bathing, without a due appreciation both of the primary operation and subsequent effects, therapeutically considered, of the means thus employed, as well as of the precise character of the disease, is not a valid one. More than this is due by the physician to his patient. To recommend or assent to the use of a new remedy or a new plan of treatment, without making one's self master of what was previously known on the subject, or, at any rate, of the general results that may be expected, is worse than empiricism; for this does plead some analogous antecedents in its favor. We dare not say how many physicians, who send their patients to a mineral spring merely because it has acquired vogue, or has become a place of fashionable resort, come under this censure. The reputation of a mineral water ought to rest on something more positive than traditional or oral evidence; and yet, in many instances, it is sustained by little else than this. One season at the springs is but the echo of another, and of the crowd who resort to them from mingled motives of health and pleasure, how few come away with a clear perception of the influence which the use of the waters exerted on them, either for good or for evil! Many, perhaps we might say the majority of the invalids who visit these places, do so often in ignorance of their real malady, and with very vague notions of the efficacy of the water; which, notwithstanding, they set about drinking as if for a wager or in fulfillment of a vow. Nor can we attach much importance to the opinion of a medical man, whose experience is limited to that gained by the stay of a week or two at the spring, and whose attention had not previously been directed to the entire subject of the therapeutical effects of the different kinds of mineral waters. Time is not given him to study their operation on himself, if he be the invalid, and still less on others, of whose antecedent history he is ignorant, and whose treatment by the use of the waters he has not had an opportunity of superintending nor of becoming minutely and methodically acquainted with it through the means of a third party.*

We commend the following observations of Dr. Moorman, which are evidently the result of experience and clinical study :—

"Some mineral waters, by varying the method of their administration, or by the interposition of appropriate adjuvants, are capable of extensive and valuable modified actions and effects upon the human body. The White Sulphur is susceptible of as many varied, different, and modified actions upon the system generally, and upon its particular organs, by varying the methods of using it, as is *mercury*, or *antimony*, or any of our leading therapeutical agents. For instance, it can be so used as to *stimulate* distressingly; or, without any *appreciable stimulating effects*. It can be so given as almost invariably to *purge actively;* or, without lessening the quantity producing such effect, but merely by changing the time and manner of taking it, it can be so given as to exert little or no cathartic operation. It may be directed to, or restrained from, the

* Bell, *ut supra*.

*kidneys*, or skin; and what, in a general way, is far more important, it can be so used as to *lie quietly* in the system, producing no excessive action upon any of the organs, and, with a quiet but sure progress, go on breaking up the obstructions in the glandular organs and removing the impediments to the proper discharge of their functions: equalizing the circulation, removing chronic inflammations, and generally restoring the energies of the system."

The effects of the *White Sulphur Water* upon the human body are represented by the author to resemble mercury in several respects; as, for instance, in its producing salivation under peculiar circumstances, and its requiring a removal of a phlogistic condition of the system before its proper and alterative effects can be obtained. "Changing from spring to spring," merely for change sake, and without allowing time for the water of any one spring to produce its best effects, or without reference to its operation on the system, meets with the merited animadversions of the author. The combined use of medicines and of different mineral waters, is the subject of another chapter. It is one, as the author justly remarks, of primary importance, but which may be summed up in a few words. As a general thing, our chief, and in the great majority of cases of chronic disease, those to which alone mineral waters are adapted, our sole reliance will be on their use during the time of the invalid's sojourn at the springs. If an invalid is still suffering from the remains of an acute disease, or of a phlogosis, his system must be brought down to the line of chronicity by suitable therapeutical treatment, or if acute symptoms supervene on chronic ones during the drinking of the waters, their use must be suspended, and the usual remedies directed in the same manner as if the patient were at his own home. But when we have to deal with a chronic disease, in which the waters of a mineral spring are both tolerated and act on the organs in a marked manner, we ought to withhold all the ordinary pharmaceutical agents, and allow medical hydrology full time and opportunity for the display of its benign effects. If the desired relief is not obtained, or if it is thought to be necessary to act on organs which are not impressed by the waters of the spring at which the invalid is staying, then comes the time for a change to be made, not through caprice or impatience, but for reasons understood and assigned by the resident and prescribing physician. By judicious changes made under the advice of a competent and experienced observer of this class, the invalid might realize all the benefits of the successive or alternate use of a series of mineral springs. Thus, for example, the treatment of a case of visceral congestion might be begun by drinking the water of a spring of the saline aperient class, and continued by that of a sulphureous and then a chalybeate water, with occasionally the judicious interposition of a gaseous one. A course of this kind would be in harmony with that pursued in the treatment of most diseases by ordinary therapeutic treatment. It would secure, moreover, a distribution of invalids among the different springs, and additional variety and change of scene, with fresh inspiring hopes, in thus going from one spring to another.

These views are well presented by Dr. Moorman, both under the head above mentioned and also under that of "Prescribing Mineral Waters." He shows a proper discrimination of the distinctive remedial properties of each spring, and, consequently, warns against the extreme and extravagant eulogies of any one.

We are not able, in the small space allotted to us, on the present occasion, to follow the author on the early history, topography, and chemical constituents of the several springs of which he speaks,—beginning with the celebrated White Sulphur. His experience of the circumstances under which invalids derive most benefit from the use of the waters of this spring, by drinking and bathing, is entitled to great deference, if not entire adoption. On one point he lays great stress, viz.: the necessity, in certain cases described by him, of letting the sulphuretted hydrogen gas escape from the water before it is drunk. We shall not follow him in his arguments in favor and explanation of his practice in this particular; but would merely remark, that he shows good cause for the distinction which he makes between the effects of the gaseous and non-gaseous water of the White Sulphur Spring. Much of what is said in the volume before us, under the head of "General directions for the use of the White Sulphur water," was anticipated in a previous chapter on "Mineral waters in general." In fact, if we are thoroughly acquainted with the principles which ought to govern in directing the use of the entire class, we shall have little embarrassment in dealing with each separate spring—as regards time, dose and repetition of dose, and hygienic rules to be observed by the invalid at the springs.

Dr. Moorman enumerates the diseases to which the White Sulphur waters are more immediately adapted, and adds a few succinct directions for their appropriate use. In dyspepsia, in which, when it has lasted some time, the author, following the general Southern belief, tells us that we almost invariably find the biliary secretions either vitiated in quality or deficient in quantity, this water has long maintained a high character. We may dispute the soundness of his pathological explanation, while admitting the facts furnished by his experience of the relief and cure, also, of this troublesome malady, after a suitable course at the White Sulphur Springs. He cautions against the mistaking of primary effects for a permanent and constitutional change, which is the necessary condition for recovery, and which requires a longer time and more perseverance in the treatment than invalids, who already, after a short trial, think themselves well, are inclined to give. "Chronic Gastro-Enteritis, or Irritation of the Mucous Membrane of the Stomach and Bowels," is believed by the author, and not without good reason, to be the disease, with its multifarious symptoms and concomitants, under which the largest class of invalids who visit our mineral springs suffer. The causes are readily found in excessive alimentation, the drinking of wine and liquors, drugged often by poisonous substances, fast eating, chewing and smoking tobacco, and the immense consumption of strong coffee, on the one hand, and an incessant strain on the brain, in all the divisions of labor and pursuit after wealth and political preferment, on the other. Associated with intestinal derangement in the female, are leucorrhœa and chlorosis, with the not unfrequent concomitant of hysteria.

"Diseases of the Liver" naturally engage the attention of Dr. Moorman, whose observations on their pathology, and the adaptation of sulphur water to their different stages and complications, merit careful perusal. In *costiveness* and in *hemorrhoids*, this water displays considerable efficacy. "Incipient calculous affection is relieved by the water, pretty much in proportion as it corrects the digestive and assimilative functions, improves the blood, and brings the general economy into a natural type, preparing the kidneys to resist foreign encroachments upon their functions, and to elaborate, from healthy blood, proper and healthy secretions." Diseases of the female sexual organs, chronic affections of the brain, nervous diseases, are passed in review, and the particular conditions under which the White Sulphur water may be used in them with advantage, are stated by the author. While warning against the employment of the White Sulphur water in *tubercular phthisis*, Dr. Moorman points out its good effects in what he calls *sympathetic consumption*, and in *bronchitis*. Consistent with his dominant pathology, that the chronic derangement of the function of any organ extends itself to others, Dr. Moorman thinks that in the restoration of the lost balance, and especially of the functions of nutrition and the secretions to their healthy rhythm, must we look for a removal of the predominant and evident disease. Taking an enlarged view of this kind, respecting the etiology and connections of skin diseases, he finds an explanation, and, we believe, a true one, of the great benefit in them by drinking the sulphur water, and its use conjointly as a bath. *Rheumatism*, *gout*, *dropsy*, *scrofula*, in certain forms and stages and under specified circumstances, find, in many instances, their cure in the sulphur water. *Mercurial diseases* and *secondary syphilis*, are, in the author's opinion, more certainly removed than other morbid conditions which we are called upon to combat by recourse to the White Sulphur water. If we refer to what is said of "Diseases of the Heart," in connection with sulphureous water therapeutics, it is to repeat the warning of the author against this agent in hypertrophy of the organ. He takes care to discriminate between organic disease and sympathetic affections of the heart, in many of the latter of which the sulphur water is beneficial.

Dr. Moorman next directs our attention to the situation and chemical composition and remedial value of the other Virginia Springs, in the region of the White Sulphur ones. Opportunity is not allowed us to give much more than the titles of these health-conferring fountains, nor to repeat the appreciative notices of them in the volume before us. With a family resemblance of the sulphureous springs to each other, there are, however, distinctive traits belonging to each, which entitle them to a separate study as therapeutic agents. Among these we may mention the Salt Sulphur, the Red Sulphur, the Blue Sulphur, the Roanoke Red Sulphur, Daggar's or Dibrell's, the New-River White Sulphur, and the Montgomery White Sulphur Springs. In reference to the reputed effects of the water of the Red Sulphur Springs, in pulmonary affections with irritative fever, we could have wished that Dr. Moorman had adduced more recent and enlarged experience, in addition to the over-sanguine representations made by the late Dr. Hunt, of Washington City. The efficacy of the Salt Sulphur water as a more active cathartic than any other one "of the geological region in which it exists," while at the same time it is neither harsh nor violent, is displayed in that form of dyspepsia in which constipation is a leading and troublesome symptom. "This water, like all our sulphur waters, will sometimes distinctly reduce the frequency and force of the pulse," not, however, by any direct sedative action upon the circulation, but by its deobstruent operation on the viscera generally, in restoring and increasing the secretions. The Salt Sulphur Iodine Spring, in the vicinity of the one just noticed, has attracted attention of late years, and has been used in various glandular and other affections, —rheumatic, syphilitic, and chronic cutaneous, in which iodine has been found serviceable.

The Thermal Springs of this region furnish all desirable balneological varieties for the necessities and tastes of invalids. The "Warm Spring," with its glorious bath and a temperature adapted to a vast majority of those who can indulge in warm bathing, merits, if merely as ministering to a luxurious indulgence, a visit from remote parts of the Union. The "Hot Springs," a few miles distant, give also, in their different temperatures, means for warm as well as hot and vapor bathing. Still of the thermal class, is the water of the Sweet Springs, but of just such a reduced temperature, 73° F., as adapts it to a large class of bathers, whose systems soon react after the first slight shock of immersion. Under the same head we must rank the Holston Spring, the water of which is of a temperature of 68·5° F., and also the Red Sweet Springs, of a temperature of 75° to 79° F.

The "Sweet Springs" and the Holston belong to the mild saline class, a more active example of which is furnished in the Alleghany Spring, in the County of Montgomery, near the Virginia and Tennessee Railroad.

Another and somewhat peculiar class of saline springs, in this region, is that known commonly under the title of "Alum Springs," the waters of which contain, in addition to the ordinary salines, chalybeates, and also aluminous salts, and an excess of sulphuric acid. The chief of these are the Rockbridge and the Bath, Pulaski, and New London Alum Springs, and one of Stribling's, and the Yellow Springs. In quite another region, or in Lower Virginia, at Richmond, is the Church Hill Alum Spring. Of the simple chalybeate water, Rawley's Spring furnishes the most active. Next will come the "Rockbridge Baths," equidistant from Lexington and Goshen Depot, on the Great Central Railroad.

In the northeastern portion of Virginia, within a few miles of the Potomac and near the ridge of the North Mountain, are the Bath, or "Berkeley Thermal Springs," (72° to 74° F.) These Springs exchange visitors with the Bedford Springs of Pennsylvania, from which they are distant only a day's ride. In a southeast direction from the Berkeley, and at the foot of the Blue Ridge, on the other side of the great valley, are the saline "Shannondale Springs," on the line of the railroad from Harper's Ferry to Winchester, and five miles distant from the latter place is "Jordon's White Sulphur." Continuing up the valley and on the road to Staunton, we meet with "Bonner's Springs" or the Seven Fountains—blue and white sulphur, chalybeate, freestone, slate and limestone, and willow. A road branching off from Winchester takes us to the Capon Springs, with their fine body of water and good air and good cheer for those who visit them. Below the Blue Ridge, and near the branch railroad from Alexandria to Warrentown, in Fauquier County, are the White Sulphur Springs. Fifty miles west of Lynchburg are the Colyer's White and Black Sulphur Springs, and in the County of Mecklenburg, near the town of Clarkville, are the sulphureous saline Buffalo Springs.

In Kentucky we find the celebrated "Blue Lick Sulphur Springs," also the Harrodsburg, the Rochester, and the Olympian. Of the springs of the Southern States we can only allude to the thermal ones of Buncombe, North Carolina, (94° to 104° F.,) and those of Washita, in Arkansas, (145° to 200° F.)

Among the mineral springs of the West proper, a knowledge is opened to us of an attractive spot, with an effective mineral spring, called the "Ohio White Sulphur," on the banks of the Scioto River, in Delaware

County, eighteen miles from Columbus. Improvements and decorations of the grounds, and the construction of houses on a large scale, with the adjuncts of baths of various kinds, have been recently made by the liberal proprietor, Mr. A. Wilson. Just such a spot was wanted on the great route of travel between the East and the West, where people could meet from those opposite parts of the Union, for health and pleasure, and, it may be, as a middle ground for the easy adjustment of business matters, relieved from the dryness of detail and association in store and warehouse.

Quite a useful feature in Dr. Moorman's volume is a map and synopsis of the different routes and distances from the Atlantic seaboard to the Springs south and west of the Potomac. A colored plan of the grounds around the White Sulphur Springs is also given.

In conclusion, we can have no hesitation in regarding this work as one filled with instructive observations and advice, which both physicians and invalids may turn to good account. It is a Manual of the Virginia Springs, with which a visitor to any one of them can hardly dispense, and the perusal of which, while guiding the medical adviser in the course he should direct his patient, will also save the latter himself from hazardous, and not seldom dangerous and empirical experiments, and teach him that while it is in his own power to ward off diseases from himself, he must leave to the specially instructed and experienced the task of curing them.

The scope and prominent heads of the subjects of Dr. Bell's treatise on Baths may be pretty well understood from the title-page. The observation made in the preface of the first edition of this work applies, we believe, to the literature of the subject at the present time. It is, that until the appearance of Dr. Bell's work on Baths and Mineral Waters, in 1831, there was no one in the English language in which the different kinds of baths, cold, warm, hot, vapor, and sea, were severally considered, and their resemblances and contrasts, and their successive and alternate uses, pointed out. "Each kind of bath was considered too much in itself, and as a consequence, without its due relation to the others." Of the manner in which the author has handled the various topics successively brought up for examination, we are the less required to speak on the present occasion, after the unstinted praises lavished on the first edition of this treatise, on both sides of the Atlantic.* It commends itself to all classes of readers, both for its giving the history of bathing among the

---

* "The work of Dr. Bell is the most complete monograph, we do not hesitate to say on the subject, that has ever issued from the press, and should find a place, as a work of reference, in the library of every practitioner."—*British and Foreign Medico-Chirurgical Review*, April, 1851.

nations of antiquity, as well as among those in nearly all parts of the world at the present time, and for the connected views which it offers of the dietetic and medicinal uses of water. It displays to the reader a wide range, from the extent and magnificence of the Roman baths to the extemporized vapor bath of the American Indian, made of wicker-work covered at the top by skins. Ten chapters are taken up with details of the "Watery Regimen," in what relates to the internal use of water in health and in disease, as gathered from the ancient writers, and from the Arabian, Spanish, Italian, English, French, and German ones. Among the English writers on the watery regimen more particularly quoted, are Floyer and Baynard. The different kinds of water are described, and the means of purifying that which is bad is given. Hydropathy in its rational features takes up three chapters. After this we find treated, in regular succession, the hygienic and therapeutical operation of Cold and Sea Bathing, the latter of which is the subject of four chapters. To these succeed five others on Warm Bathing, and two on the Hot Bath. Six chapters are appropriated to Vapor Baths, including the dry and the moist, simple and medicated. Pulmonary Atmiatry, including the inhalation of artificial atmospheres and iodine inhalations, also those of ether and chloroform, receives a share of notice. Under the head of Medicated Baths we noticed those of Mineral Springs as the chief ones, then Mud Baths, Iodine and Chlorine ones, and those of the Mineral Acids, and of Compressed Air. A notice of a Common Air Bath closes the last or fifty-sixth chapter of the volume.

After no little experience and observation directed to the subject, we are free to say, that in no division of the Materia Medica is there such a want of knowledge, and such a manifestation of empiricism by physicians generally, as in the case of the different kinds of bath. Half the time, the tepid, warm, and hot baths are spoken of as warm, and quite commonly the hot is confounded with the warm, a mistake equivalent to that of telling a patient to drink rectified spirits instead of Madeira or Sherry wine. The perusal of a work like the present one would prevent these discreditable exhibitions of ignorance in members of a profession called learned, and be the means of preventing much *mala praxis* on the part of the physician, and of a saving of life on the part of the infirm and the sick.

